# Anticonvulsant Activity of *Teucrium polium* Against Seizure Induced by PTZ and MES in Mice 

**Published:** 2010

**Authors:** Mohammad Javad Khoshnood-Mansoorkhani, Mahmood Reza Moein, Narjes Oveisi

**Affiliations:** a *Department of Toxicology and Pharmacology, Faculty of Pharmacy, Shiraz University of Medical Sciences, Shiraz, Iran.*; b * Department of Pharmacognosy, Faculty of Pharmacy, Shiraz University of Medical Sciences, Shiraz, Iran.*

**Keywords:** *Teucrium polium*, Seizure, MES, PTZ, Flavonoid

## Abstract

*Teucrium polium *(Labiatae) is a plant that widely grows in Iran. Some of species of *Teucrium *are used for a considerable range of actions in traditional medicine and *T. polium *has frequently been used as anticonvulsant. In this study, we investigated the protective effects of *T. polium *ethanolic aqueous extracts and related fractions on seizures induced by pentylenetetrazole (PTZ) and maximal electroshock stimulation (MES). Moreover, presence of alkaloids, terpenoids, tannins and flavonoid contents were evaluated. It was found that aqueous extract (ED50 = 22.4 mg/kg body weight) and related *n*-butanol fraction (ED50 = 12.6 mg/kg body weight) have antiseizure effects comparing to control groups. There was no difference between preventing of PTZ-induced death and MES-induced hindlimb tonic extension (HLTE) in ethanolic extract comparing to control groups. Our results showed that the amount of flavonoid quantity present in aqueous extract is higher than that of ethanolic extract. These data also showed that the quantity of the flavonoid in *n*-butanol fraction of aqueous extract is more than other fractions. In conclusion, it was realized that flavonoid rich extracts are more potent than other fractions in showing antiseizure effects.

## Introduction

There is renewed worldwide interest in the use of plants to relieve or cure different diseases such as neurological disorders like epilepsies, which have a high incidence in the world population (1)*. *Herbs may have antiepileptic effects in several ways. Some herbs may increase brain levels and/or the binding of nerve transmitter gamma aminobutyric acid (GABA), which quiets nerve activity (2). The Labiatae (Lamiaceae) is one of the largest and most distinctive families of flowering plants, with about 220 genera and almost 4000 species worldwide (3). Because of the high rate of species diversity and endemism in Labiatae, many species are used in traditional and folk medicine in Iran (4). A wide range of compounds such as terpenoids, iridiods, phenolic compounds and flavonoids have been reported from the members of the family (5).

use in folk medicine. In Iranian folk medicine, *Teucrium polium *is used as anticonvulsant medicine (12). Of 28 compounds being identified in the essential oil of this plant with 99.75%, the combination of α-pinene (12.52%), linalool (10.63%), caryophyllene oxide (6.69%), β-pinene (7.09%), and caryophyllene (6.98%) with 46.91 percent constitute the highest percentage of essential oil. A study on oil obtained from *Teucrium polium *grown in Iran revealed the presence of sesquiterpenes as major components oil (13). The goal of this study was to investigate the anticonvulsant effect and relationship between this effect and compounds present in the plant.

## Experimental

*Animals*

Adult male NMRI mice (25-30 g) was purchased from the Animal House of Shiraz University of Medical Sciences Shiraz, Iran. The animal house temperature was maintained at 22±2ºC with a 12 h light/dark cycle. All animals were kept for one week prior to experimentation and were given free access to food and water. Each animal was tested once. All animal experiments were carried out in accordance with recommendations of the Declaration of Helsinki and internationally accepted principles for the use of experimental animals.

*Plant*

*Teucrium polium *was purchased from Karaj Plant Institute freshly, and was authenticated by M. Kamalinejad and a voucher specimen (No. 628) was deposited in the Herbarium of Pharmacy School, Shahid Beheshti University of Medical Sciences, Tehran, Iran.

*Preparation of the extracts*

For preparation of aqueous extract, mixture of dried aerial parts of plant in boiling water (1:10) was placed at 50ºC water bath for 1h and was kept at room temperature for 24 h. Then, the mixture was filtered and these stages were repeated on the residue. The filtrates were concentrated and dried to yield 16.3% crude extract.

For preparation of ethanolic extract, the mixture of dried aerial parts of plant in ethanol 96% (1 : 8) was kept at room temperature for 72 h and was filtered. These stages were repeated on the residue. The filtrates were concentrated and dried to yield 14.7% crude extract (14).

*Preparation of the fractions*

Aqueous and ethanolic extracts (30 g) were dissolved in 30 mL of water and water : methanol (1 : 1), respectively. These fractions were separated in order to increase polarity from petroleum ether, chloroform, ethyl acetate, and *n*-butanol, respectively. Residue solutions of any stages (three times for any solvent) were dried at room temperature (15).

*Behavioral tests*

Behavioral tests were performed on groups consisting 10 mices. In order to study on anticonvulsant activity, at least three different concentrations of aqueous extract, ethanolic extract, fractions of extracts and diazepam as positive control were prepared freshly. 

PTZ and aqueous extract were dissolved in normal saline (NS) while diazepam (DZP), ethanolic extract and all fractions were dissolved in 40% dimethyl sulfoxide (DMSO). Control groups received NS or DMSO.

All controls and extracts were administrated intraperitoneally (IP) in volume of 10 mL/kg animal body weight.

The time for DZP and other treatments to reach the maximum effect were determined to be 30 min after IP injection.

*Pentylenetetrazole seizure model*

Animals were treated with DZP, NS, DMSO, *Teucrium polium *extracts and fractions. Thirty min later, seizure was induced by the IP administration of 80 mg/kg of PTZ. The following parameters were recorded during the first 30 min after PTZ administration**:**

1. Latency to the onset time of myoclonic and tonic-clonic seizures.

2. Protection from HLTE (Hindlimb Tonic Extention) and death.

Cut-off time was 1800 sec (1, 16-17).

*Maximal electroshock seizure model*

Electro-convulsive shock usually induces HLTE in 99.9% of the animals. The electrical stimulus (120 V, 50 Hz, 2s duration) was applied

through ear-clip electrodes using a stimulator apparatus. Animals were treated with DZP, NS, DMSO, *Teucrium polium *extracts and fractions. Thirty minutes later, seizure was induced by electroshock and protections from HLTE were recorded (18).

*Preliminary phytochemical analysis*

The *Teucrium polium *extracts and fractions were screened for flavonoid, terpenoid, alkaloid and tannins by the previously reported methods (14, 19-22). Flavonoid contents were evaluated with “aluminum trichloride (AlCl_3_)” reagent and “rutin” as a standard (23-24).

*Statistical analysis*

The dose of the compound required for inducing anticonvulsant effect in 50% of animals and its associated 95% confidence limit were calculated by SPSS software and probit regression. Data obtained from delay convulsion behavior were expressed as Mean ± SEM and were analyzed by One-way ANOVA along with Dennett’s post test. p < 0.05 was considered significant. 

## Results and Discussion

*Behavioral tests*

The results demonstrated that the *Teucrium polium *has anticonvulsant activity in both PTZ and MES seizure models. Table 1 shows the ED_50_ with confidence limits of diazepam, aqueous extract and its fractions in HLTE induced by MES and death by PTZ models. It was found that aqueous extract and its fractions especially *n*-butanol fraction have antiseizure effects comparing to control group. No difference was found between preventing of death and HLTE in ethanolic extract and control group in both PTZ and MES models (data not shown). The aqueous extract and its fractions increased the latency of convulsion parameters induced by PTZ, but the ethanolic extract showed very small effects.

**Table 1:** ED_50_ of diazepam, aqueous extract and related fractions of *T. polium *on HLTE induced by MES and death by PTZ models in mice

Compound	**ED** **50 ** **(mg/kg) mean (lower limit** **-upper limit)**	
	**PTZ**	**MES**
Diazepam	1.6 (1.1-2.4)	5.6 (3.9-8.3)
Aqueous extract	22.4 (16.6-32.6)	560.2 (339.6-1023.1)
Chloroform fraction	78.4 (55.1-136.8)	53.9 (30.5-88.9)
Ethyl acetate fraction	142.5 (99.1-232.2)	365.1 (279.0-663.2)
*n*-Butanol fraction	12.6 (7.9-20.0)	16.7 (10.2-26.3)

**Table 2:** The amount of total flavonoid of aqueous extract *T. polium *L. and related fractions

**Compound**	**Flavonoid concentration (mg/mL)**
Aqueous extract	4.52 ± 0.28
*n*-Butanol fraction	3.05 ± 0.21
Ethyl acetate fraction	2.51 ± 0.18
Ethanolic extract	1.89 ± 0.18

**Figure 1:**
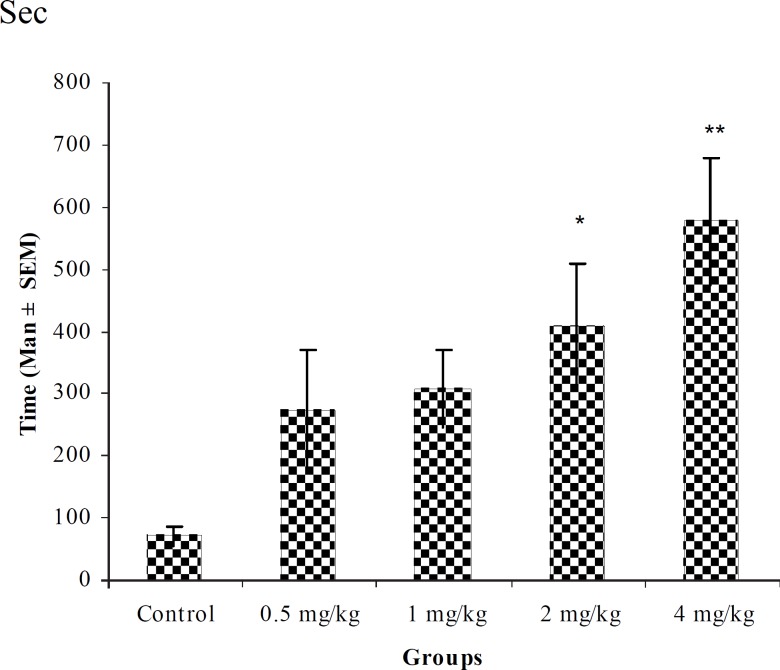
Effect of intraperitoneal injection of different doses of diazepam on myoclonic seizure onset time (sec) induced by pentylenetetrazole 80 mg/kg. (n = 10) * p < 0.05 ** p < 0.01

**Figure 2:**
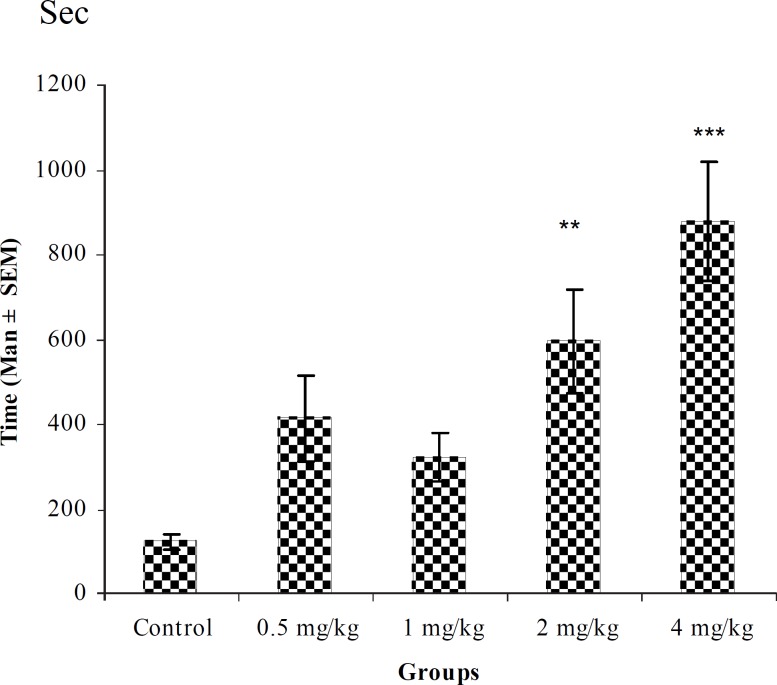
Effect of intraperitoneal injection of different doses of diazepam on tonic–clonic seizure onset time (sec) induced by pentylenetetrazole 80 mg/kg. (n = 10) ** p < 0.01 *** p < 0.001

**Figure 3:**
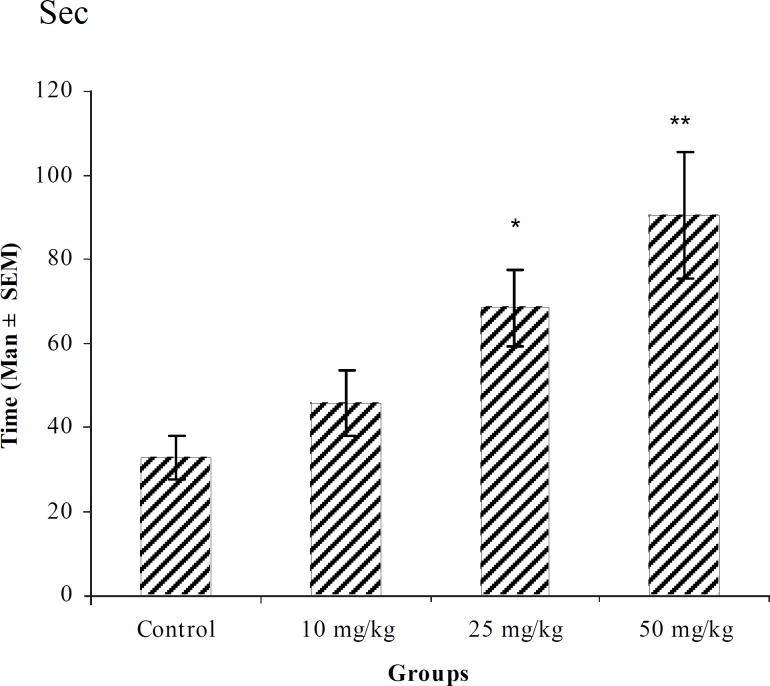
Effect of intraperitoneal injection of different doses of *Teucorium polium *aqueous extract on myoclonic seizure onset time (sec) induced by pentylenetetrazole 80 mg/kg. (n = 10) * p < 0.05 ** p < 0.01

**Figure 4:**
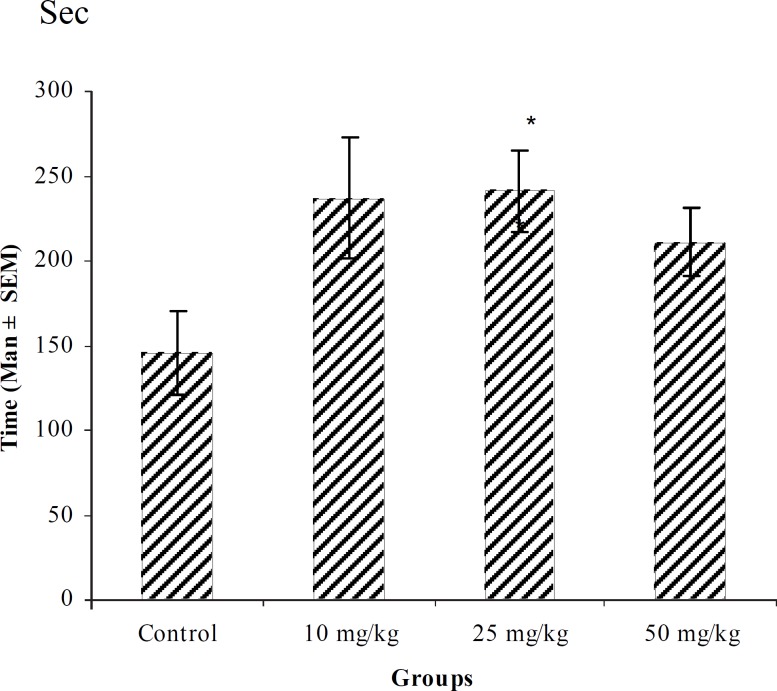
Effect of intraperitoneal injection of different doses of *Teucorium polium *aqueous extract on tonic-clonic seizure onset time (sec) induced by pentylenetetrazole 80 mg/kg. (n = 10 ).

**Figure 5:**
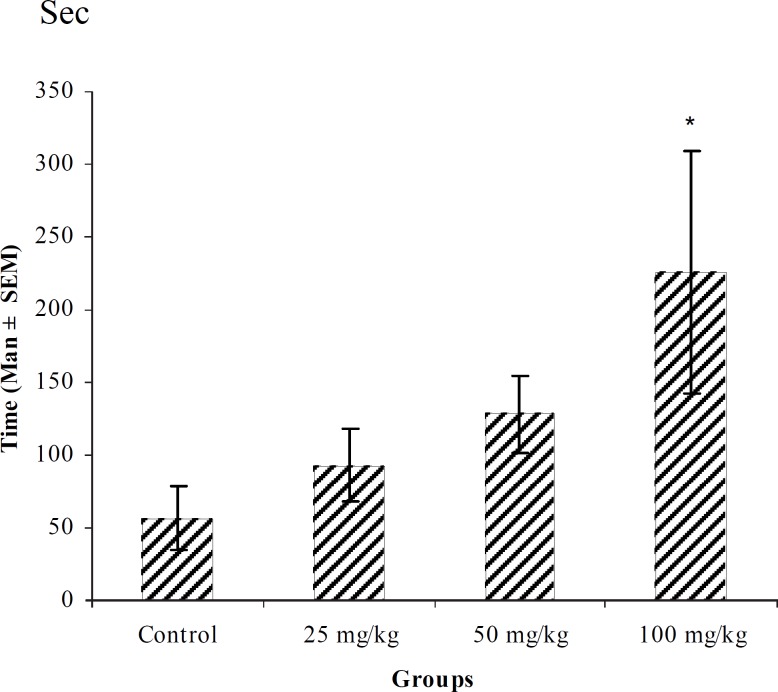
Effect of intraperitoneal injection of different doses of chloroform fraction of *T. polium *on myoclonic seizure onset time (sec) induced by pentylenetetrazole 80 mg/kg. (n = 10) * p < 0.05

**Figure 6:**
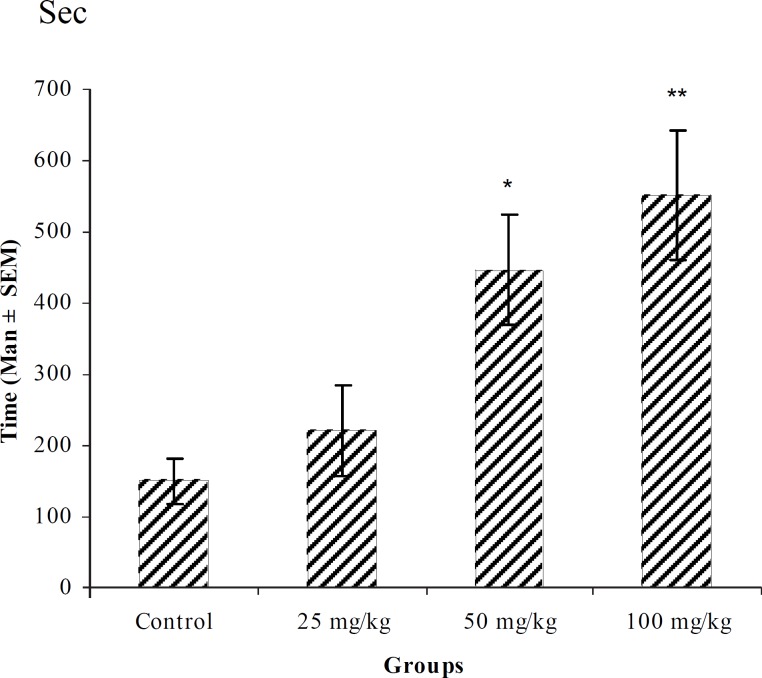
Effect of intraperitoneal injection of different doses of chloroform fraction of *T. polium *on tonic-clonic seizure onset time (sec) induced by pentylenetetrazole 80 mg/kg. (n = 10) * p < 0.05 ** p < 0.01

**Figure 7:**
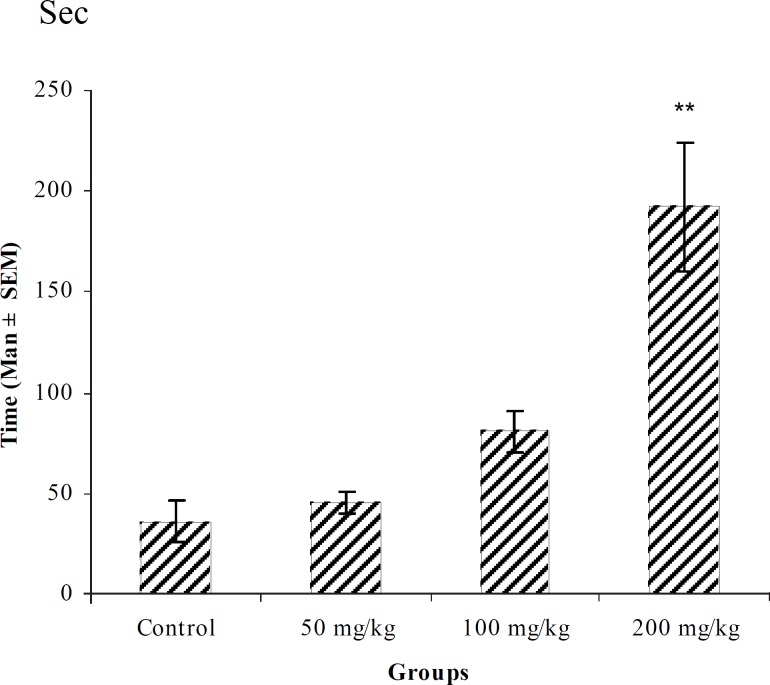
Effect of intraperitoneal injection of different doses of ethylacetate fraction of *T. polium *on myoclonic seizure onset time (sec) induced by pentylenetetrazole 80 mg/kg. (n = 10) ** p < 0.01

**Figure 8:**
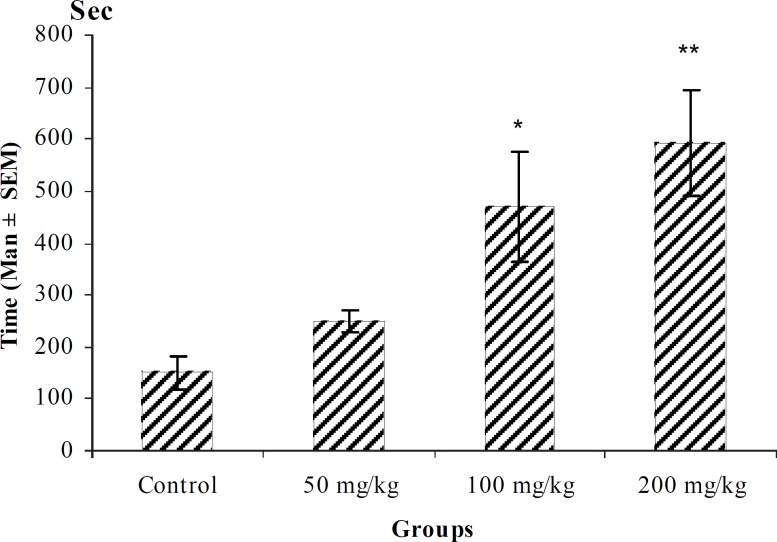
Effect of intraperitoneal injection of different doses of ethylacetate fraction of *T. polium *on tonic-clonic seizure onset time (sec) induced by pentylenetetrazole 80 mg/kg. (n = 10) * p < 0.05 ** p < 0.01

**Figure 9:**
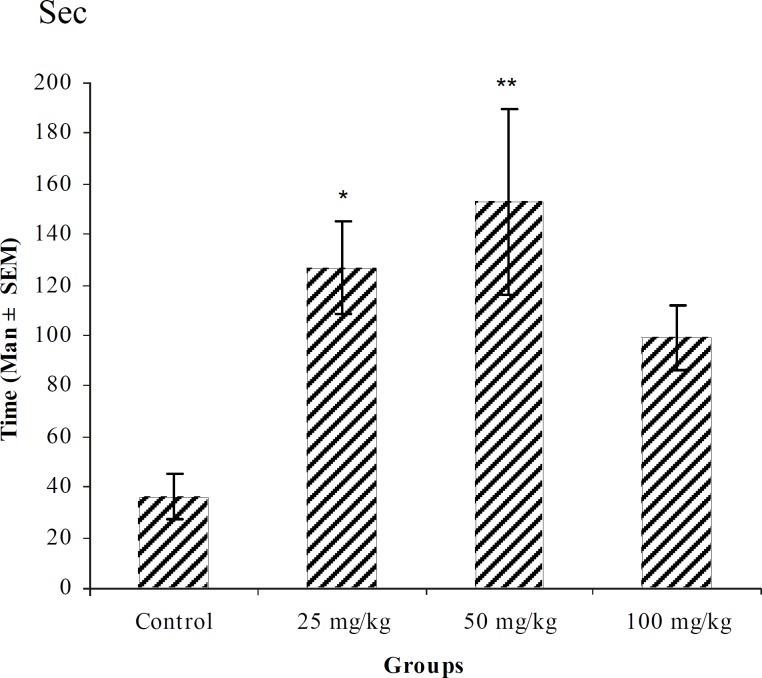
Effect of intraperitoneal injection of different doses of *n*-butanol fraction of *T. polium *on myoclonic seizure onset time (sec) induced by pentylenetetrazole 80 mg/kg. (n = 10) * p < 0.05 ** p < 0.01

**Figure 10:**
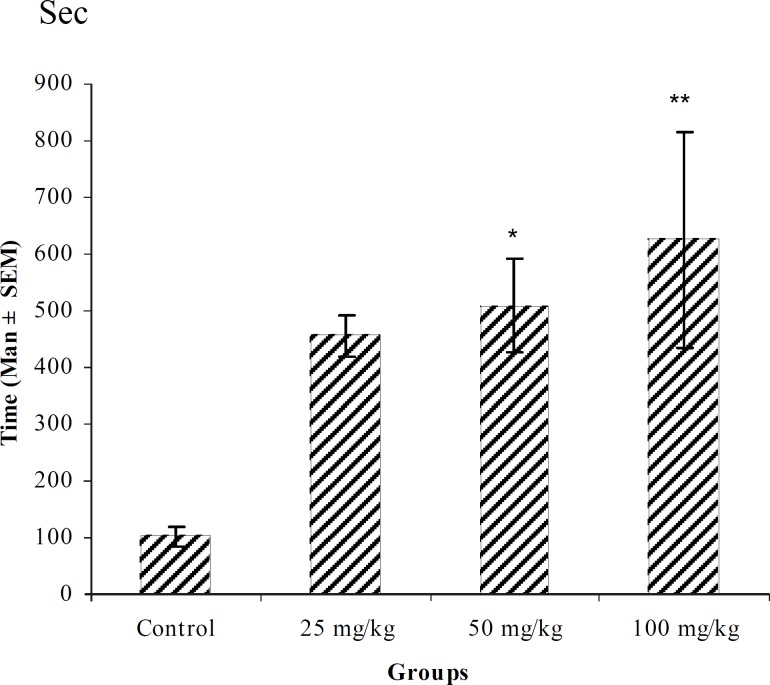
Effect of intraperitoneal injection of different doses of *n*-butanol fraction of *T. polium *on tonic-clonic seizure onset time (sec) induced by pentylenetetrazole 80 mg/kg. (n = 10) * p < 0.05 ** p < 0.01

*Preliminary phytochemical screening*

Phytochemical test showed that most of the flavonoid quantity and tannin are present in aqueous extract and related fractions, while ethanolic extract contained terpenoids.

Among the tests used for evaluation of anticonvulsant activity, the MES and PTZ tests are of predictive relevance regarding the clinical spectrum of activity of experimental compounds, since the MES and PTZ tests are assumed to identify anticonvulsant drugs effective against human generalized tonic-clonic and absence seizures, respectively (16, 18).

MES-induced seizure can be prevented either by drugs that inhibit voltage-dependent Na^+^ channels such as phonytoin, Na valproate, felbamate and lamotrigine; or by drugs that block glutamatergic receptor such as felbamate. On the other hand, drugs that reduce T-type Ca^++ ^currents, such as ethosuximide can prevent seizures induced by PTZ. Drugs that enhance gamma amino butyric acid type A (GABAA) receptor mediated inhibitory neurotransmission such as benzodiazepines and phenobarbital and perhaps valproate and felbamate can prevent this type of seizure (25).

Current available anticonvulsant drugs are able to efficiently control epileptic seizures in about 75% of the patients. Furthermore, undesirable side effects from the drugs used clinically often render treatment difficulty; so there are demands for new types of anticonvulsants drugs. One of the approaches to search for new antiepileptic drugs is the naturally occurring compounds, which may belong to new structural classes (25). In Iranian traditional medicine, the extract obtained from the aerial parts of *T. polium *was used as an antiepileptic remedy (11).

In the present study, the effect of the aqueous extract, related fractions and ethanolic extracts of *T. polium *on seizure induced by MES and PTZ in mice was evaluated and the results evidently demonstrated for the first time that the *T. polium *aqueous extract and fractions of it are able to produce potent anticonvulsant activity in both MES and PTZ seizures.

Presence of flavonoids like apigenin, cirsimaritin, terpenoid derivatives, steroids and iridoids in the *T. polium *have been reported (6-8, 20). Existence of flavonoid in *T. polium *can explain many of its beneficiary effects (8). Flavonoids, an important class of naturally compounds, have demonstrated CNS activities such as affinity for GABAA receptors and anticonvulsion effects (23, 27). Some researchers have reported anticonvulsant activity of apigenin as glucoside flavonoid of *T. polium *(8, 26). Triterpenes are reported to possess anticonvulsant activity in some experimental seizure models like PTZ and MES. Monoterpenes also have protective effects against PTZ-induced convulsions (18).

In PTZ model, aqueous extract and related fractions of *T. polium *significantly reduced death and increased onset time of seizure behavior (Figures 1-10). ED_50_ values of aqueous extract and its *n*-butanol fraction were lower than those of other compounds. On the other hand, phytochemical investigations showed that the main constituent of aqueous extract and its n-butanol fraction were flavonoids. Therefore, it seems that the antiseizure properties of *T. polium *may be related to flavonoids and unknown polar compounds present in the plant. However, the exact mechanism(s) and the active compound(s) involved in these effects will have to be elucidated in future studies. 

The lower effect of aqueous extract against MES might reasonably be explained by concentration of active compound in the extract, which was probably low for MES seizures, but at the same time sufficient to reduce PTZ seizures.

In conclusion, considering the recorded effects of *T. polium *extract in these experiments, anticonvulsant efficacy against the above mentioned seizure type in human could be suggested.
